# LUCAT1 as an oncogene in tongue squamous cell carcinoma by targeting miR‐375 expression

**DOI:** 10.1111/jcmm.15982

**Published:** 2021-03-30

**Authors:** Kai Zhang, Qibao Wang, Bo Zhong, Zuode Gong

**Affiliations:** ^1^ Center of Stomatology China‐Japan Friendship Hospital Beijing China; ^2^ Department of Endodontics Jinan Stomatological Hospital Shandong China

**Keywords:** LUCAT1, miR‐375, tongue squamous cell carcinoma

## Abstract

Emerging studies suggested that lncRNAs play a crucial molecular role in cancer development and progression. LncRNA LUCAT1 has been proved as oncogenic molecular in lung cancer, glioma, osteosarcoma, renal carcinoma and oesophageal squamous cell carcinoma. However, its roles and function mechanisms in tongue squamous cell carcinoma (TSCC) are still unknown. We showed that the expression of LUCAT1 was up‐regulated in the TSCC cells and tissues and the higher LUCAT1 expression was associated with the poor overall survival (OS). Knockdown expression of LUCAT1 suppressed TSCC cell proliferation, cycle and migration. In addition, we demonstrated that miR‐375 overexpression inhibited the luciferase activity of LUCAT1 wild‐type and knockdown LUCAT1 promoted the miR‐375 expression in TSCC cell. Furthermore, we indicated that miR‐375 expression was down‐regulated in the TSCC cell lines and tissues and the lower expression of miR‐375 was associated with poor OS. The expression of miR‐375 was inversely correlated with LUCAT1 expression in the TSCC tissues. Knockdown LUCAT1 promoted TSCC cell proliferation, cell cycle and migration partly through regulating miR‐375 expression. In summary, this study suggested the tumorigenic effect of lncRNA LUCAT1 in TSCC cells by targeting miR‐375 expression.

## 1 INTRODUCTION

Tongue squamous cell carcinoma remains the commonest oral carcinoma, usually resulted in malfunction of speech, deglutition and mastication.^1^, ^2^, ^3^, ^4^ TSCC is famous for the ability of high proliferation and metastasis.^5^, ^6^, ^7^, ^8^ Despite recent progression in therapeutic methods such as surgery, chemotherapy and radiotherapy, the prognosis of TSCC is still under dissatisfaction.^9^, ^10^, ^11^ Thus, it is crucial to study molecular mechanism of TSCC and identify new therapeutic methods for the development of TSCC.

Long non‐coding RNAs (lncRNAs) are a group of non‐coding RNAs with longer than 200 nucleotides in the length which have no or limited protein‐coding capacity.^12^, ^13^, ^14^, ^15^, ^16^ Increasing studies suggested that several lncRNAs are found to be regulated in several tumours including gastric cancer, lung cancer, hepatocellular carcinoma, gallbladder cancer, gliomas, colorectal cancer and osteosarcoma and also TSCC.^17^, ^18^, ^19^, ^20^, ^21^, ^22^, ^23^ Recently, studies also found that lncRNAs play crucial roles in a lot of cell biological processes such as cell development, growth, apoptosis, invasion and migration.^24^, ^25^, ^26^, ^27^, ^28^ Recently, a novel lncRNA LUCAT1 was reported to be up‐regulated in several tumours such as lung tumour, glioma, osteosarcoma, renal carcinoma and oesophageal squamous cell carcinoma.^29^, ^30^, ^31^, ^32^, ^33^ For example, Sun et al^33^ indicated that LUCAT1 expression was overexpressed in lung tumour tissues and LUCAT1 knockdown suppressed the cell growth in vivo and in vitro. Gao et al^32^ found that the expression of LUCAT1 was up‐regulated in the glioma cells and samples. Knockdown expression of LUCAT1 decreased the glioma cell invasion and proliferation partly through regulating the miR‐375 expression. However, its roles, expression and function mechanisms in TSCC are still unknown.

We indicated that LUCAT1 expression was up‐regulated in the TSCC cell lines and tissues and the higher LUCAT1 expression was associated with the poor overall survival. Knockdown expression of LUCAT1 suppressed TSCC cell proliferation, cell cycle and migration.

## 2 MATERIALS AND METHODS

### 2.1 Tissues, cell transfection and culture

Fresh TSCC samples and matched non‐cancerous samples were collected from TSCC cases in Jinan Stomatological Hospital, China. All samples were immediately frozen in the liquid nitrogen after surgery. Written informed consent was obtained from each TSCC patient, and our study was approved by the clinical Institutional Ethics Committee of Jinan Stomatological Hospital. Normal keratinocyte cell (NHOK) and human TSCC cell lines (SCC4, UM1, Cal 27 and SCC1) were purchased from the cell bank of Chinese Academy of Sciences from Shanghai (Shanghai, China). Cells were kept in Dulbecco's modified Eagle's medium (DMEM) supplemented with FBS and penicillin and streptomycin. LUCAT1 siRNA and siRNA control plasmid, miR‐375 mimic and scramble, miR‐375 inhibitor and control plasmid were purchased from GenePharma Company (Shanghai, China) and were transfected to the cells by using Lipofectamine 2000 (Invitrogen, Carlsbad, CA, USA).

### 2.2 Quantitative RT‐PCR

The isolation of RNA from samples or cells was extracted by using TRIzol Kit (Invitrogen) following the instruction. The expression of LUCAT1 and miR‐375 was measured using the SYBR Green Kit (Takara, Dalian, China) on the Q5 Real‐Time PCR System (Bio‐Rad, Berkely, CA). The relative expression level of LUCAT1 and miR‐375 was determined using the ^2‐^DDCT method. The primers utilized in this study were shown as follows: GAPDH, Forward 5’‐CTCCAGTACCTACCTTACAGGGATT‐3’ and Reverse 5’‐GCTGCTGGCACCTCCA‐3’. LUCAT1, Forward 5’‐GCTCGGATTGCCTTAGACAG‐3’ and Reverse 5’‐GGGTGAGCTTCTTGTGAGGA‐3’; U6, Forward 5’‐CTCGCTTCGGCAGCACA‐3’ and Reverse 5’‐AACGCTTCAGGAATTTGCGT‐3’.

### 2.3 Cell growth, cell cycle assay and migration assay

Cell growth was determined by using CCK‐8 (Cell Counting Kit‐8) assay (Dojindo, Japan). Cells were cultured in the 96‐well plate, and the cell proliferation was measured at the 0, 24, 48 and 72 hours. Ten μL CCK‐8 solutions were added to each well, and the OD (optical density) was determined at 450 nm. For cell cycle analysis, cell was harvested and then washed with TBST for three times. Cells were then stained with propidium iodide (PI) supplemented with the ribonuclease A for a half‐hour at the room temperature. Sample was determined by FACScan flow cytometer (FACSCalibur, Mountain View, CA, USA). For cell migration assay, a scratch of cell monolayers was made with pipette tip (Eppendorf). Cells were cultured in DMEM with FBS. Wound healing capacity was measured by the microscopy at 0, 24, 48 and 73 hours.

### 2.4 Statistical analysis

Data were listed as the mean ± SD (standard deviation). One‐way ANOVA or Student's *t* test (two‐tailed) was used to detect the data with SPSS 18.0. *P* value < 0.05 was accepted as statistically significant.

## 3 RESULTS

### 3.1 LUCAT1 expression was up‐regulated in TSCC cell lines and tissues

**FIGURE 1:**
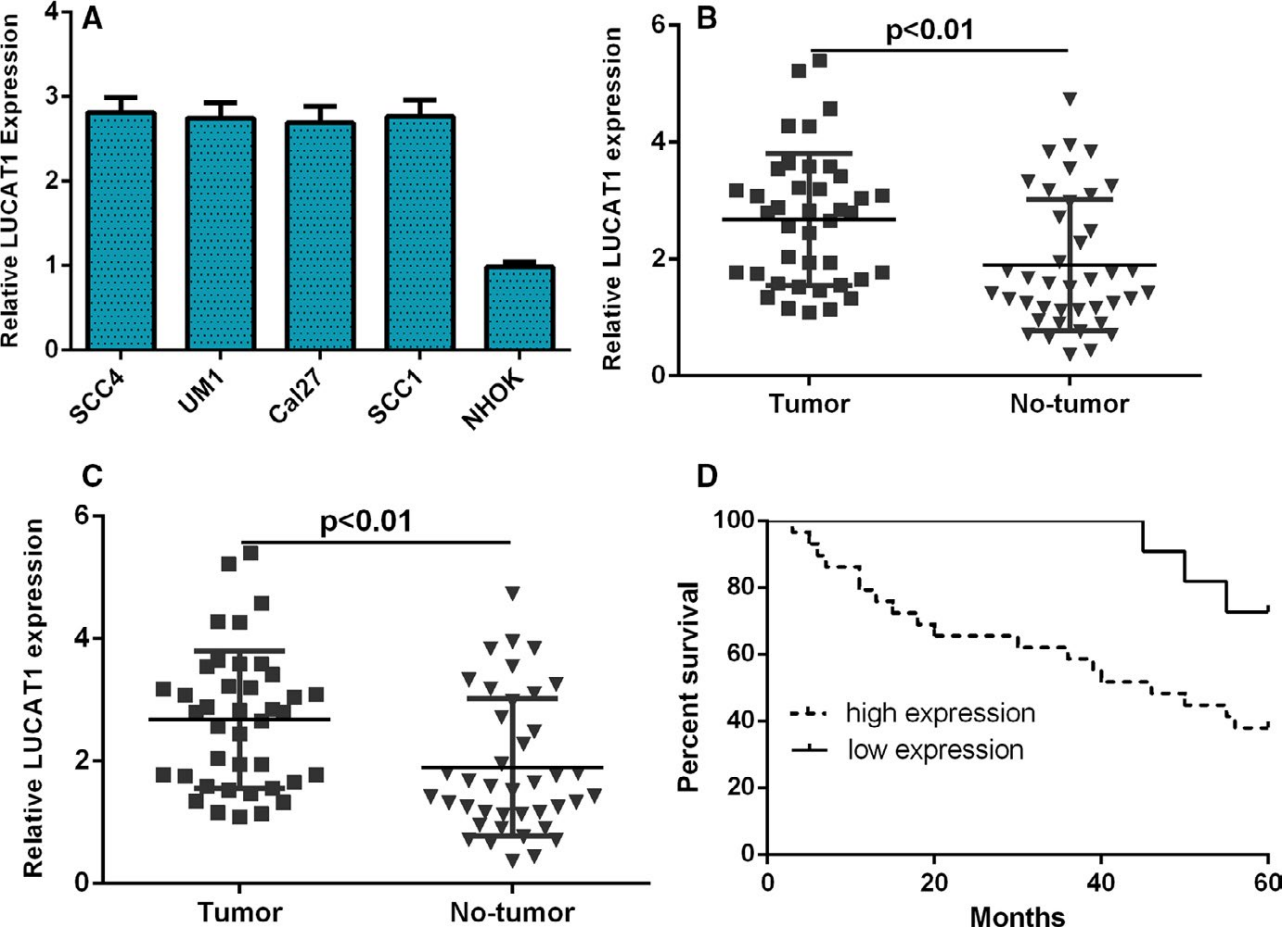
LUCAT1 expression was up‐regulated in the TSCC cell lines and tissues. (A) The expression of LUCAT1 in the TSCC cell lines (SCC4, UM1, Cal27 and SCC1) and normal keratinocyte cell (NHOK) was determined by qRT‐PCR analysis. U6 was used as the internal control. (B) The LUCAT1 expression in the TSCC tissues and adjacent normal tissues was measured by qRT‐PCR assay. U6 was used as the internal control. (C) The expression of LUCAT1 was up‐regulated in 29 patients (29/40, 72.5%) compared with adjacent normal tissues. (D) The higher LUCAT1 expression was associated with the poor overall survival (OS) among the TSCC cohort by using Kaplan‐Meier survival analysis

To explore the clinical importance of lncRNA LUCAT1 in TSCC, we measured the LUCAT1 expression in TSCC cell lines and samples using qRT‐PCR. We firstly revealed that the expression of LUCAT1 was up‐regulated in the TSCC cell lines (SCC4, UM1, Cal27 and SCC1) compared with normal keratinocyte cell (NHOK) (Figure 1A). Next, we showed that the LUCAT1 expression was higher in the TSCC tissues than in the adjacent normal tissues (Figure 1B). In addition, we found that the expression of LUCAT1 was up‐regulated in 29 patients (29/40, 72.5%) compared with adjacent normal tissues (Figure 1C). Furthermore, we indicated that the higher LUCAT1 expression was associated with the poor overall survival (OS) among the TSCC cohort by using Kaplan‐Meier survival analysis (Figure 1D).

### 3.2 Knockdown expression of LUCAT1 suppressed TSCC cell proliferation, cell cycle and migration

**FIGURE 2:**
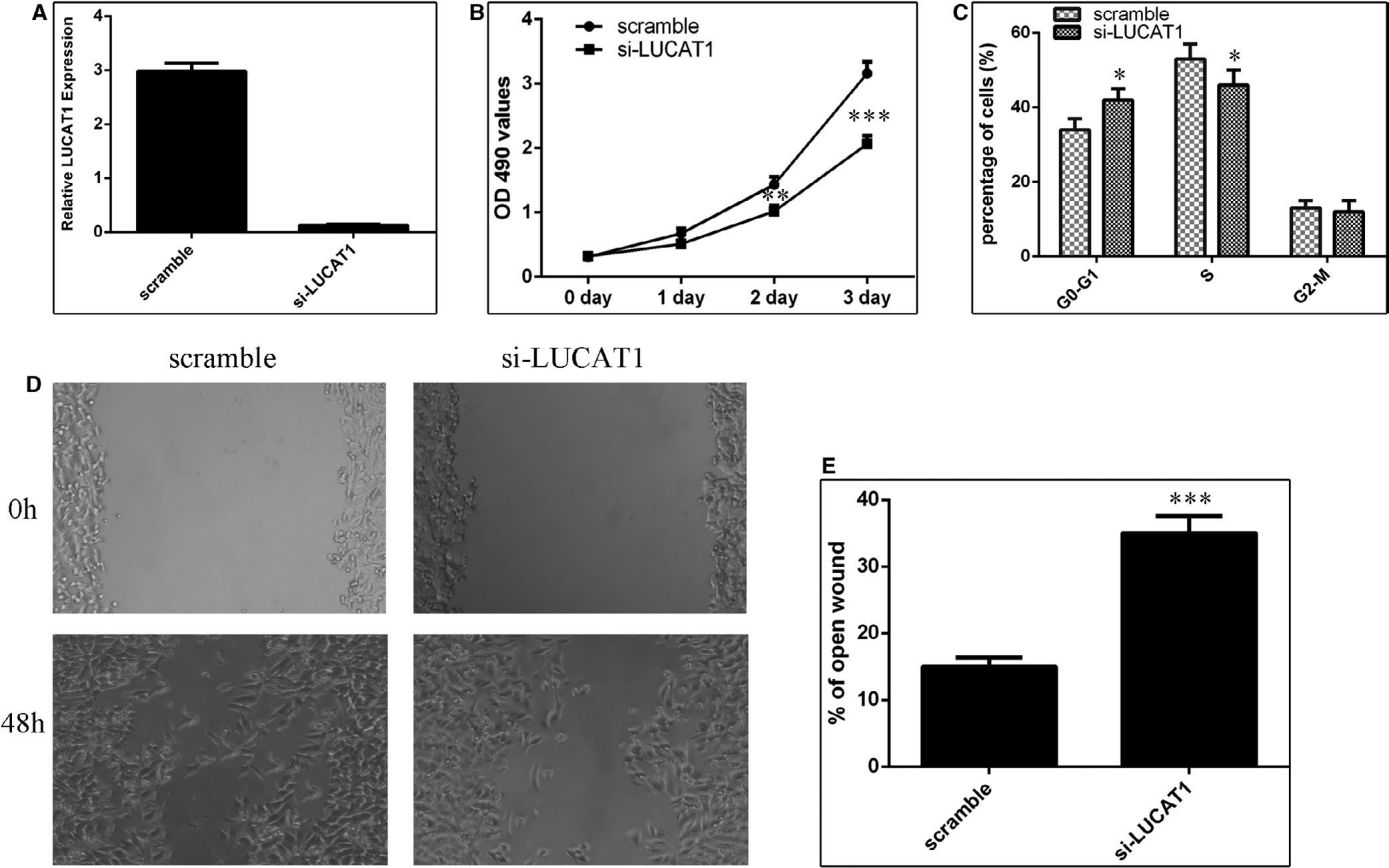
Knockdown expression of LUCAT1 suppressed TSCC cell proliferation, cell cycle and migration. (A) The expression of LUCAT1 in the SCC1 cell was measured by qRT‐PCR. (B) Knockdown expression of LUCAT1 suppressed the SCC1 cell proliferation by using MTT analysis. (C) Knockdown expression of LUCAT1 decreased the S phases in the SCC1 cell. (D) LUCAT1 silencing decreased the SCC1 cell migration using wound scratch assay. (E) The relative open wound was shown. *P < 0.05, **P < 0.01 and ***P < 0.001

TSCC cell line SCC1 cell was transfected with si‐LUCAT1 and negative control siRNA (scramble) by using Lipofectamine 2000 (Figure 2A). Knockdown expression of LUCAT1 suppressed the SCC1 cell proliferation by using MTT analysis (Figure 2B). Moreover, inhibition expression of LUCAT1 decreased the S phases in the SCC1 cell (Figure 2C). Wound scratch assay revealed that LUCAT1 silencing decreased the SCC1 cell migration (Figure 2D).

### 3.3 LUCAT1 regulated the miR‐375 expression in TSCC cell

**FIGURE 3:**
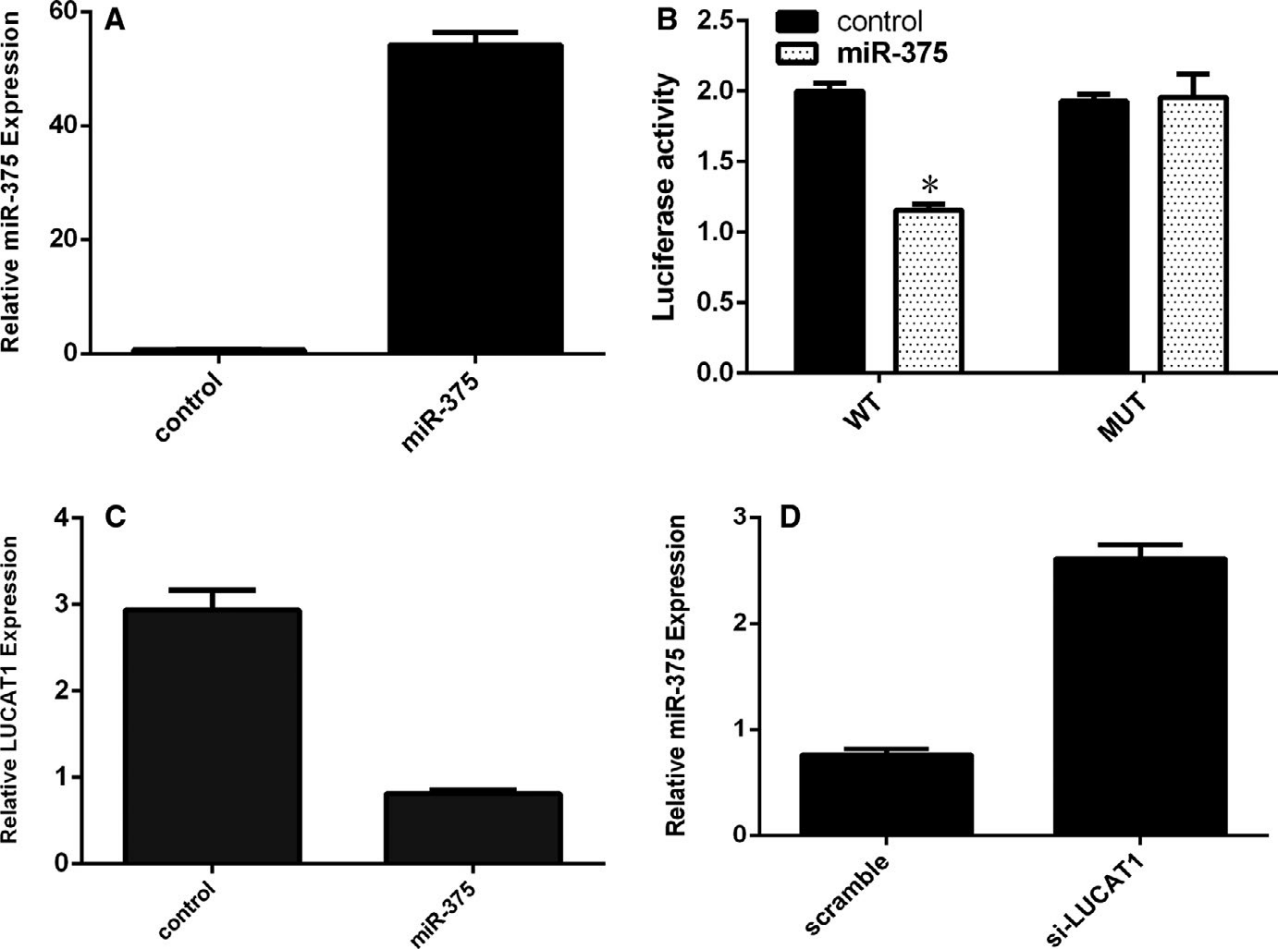
LUCAT1 regulated the miR‐375 expression in TSCC cell. (A) The expression of miR‐375 was determined by qRT‐PCR. (B) Overexpression of miR‐375 decreased the luciferase activity of LUCAT1 wild‐type but not the LUCAT1 mutant type. (C) Ectopic expression of miR‐375 decreased the LUCAT1 expression in the SCC1 cell. (D) LUCAT1 silencing promoted the miR‐375 expression in the SCC1 cell

Previous study showed that LUCAT1 could target the miR‐375 expression. TSCC cell line SCC1 cell was transfected with miR‐375 mimic and negative control by using Lipofectamine 2000 (Figure 3A). Luciferase reporter assays showed that overexpression of miR‐375 decreased the luciferase activity of LUCAT1 wild‐type but not the LUCAT1 mutant type (Figure 3B). Moreover, ectopic expression of miR‐375 decreased the LUCAT1 expression in the SCC1 cell (Figure 3C). Furthermore, LUCAT1 silencing promoted the miR‐375 expression in the SCC1 cell (Figure 3D).

### 3.4 miR‐375 expression was down‐regulated in TSCC cell lines and tissues

**FIGURE 4:**
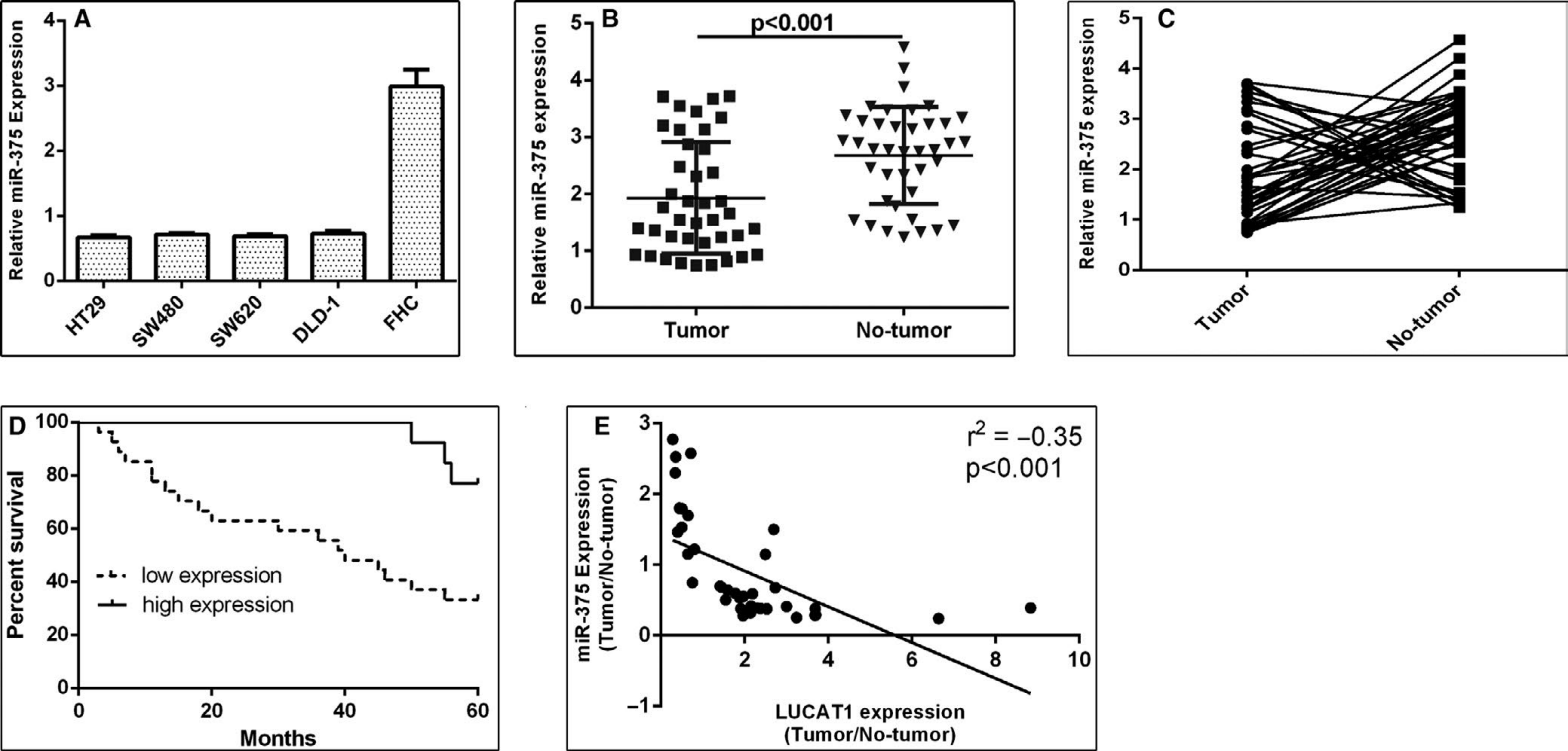
miR‐375 expression was down‐regulated in the TSCC cell lines and tissues. (A) The expression of miR‐375 in the TSCC cell lines (SCC4, UM1, Cal27 and SCC1) and normal keratinocyte cell (NHOK) was determined by qRT‐PCR analysis. U6 was used as the internal control. (B) The miR‐375 expression in the TSCC tissues and adjacent normal tissues was measured by qRT‐PCR assay. U6 was used as the internal control. (C) The expression of miR‐375 was down‐regulated in 228 patients (28/40, 70%) compared with adjacent normal tissues. (D) The lower LUCAT1 expression was associated with the poor overall survival (OS) among the TSCC cohort by using Kaplan‐Meier survival analysis. (E) The expression of miR‐375 was inversely correlated with LUCAT1 expression in the TSCC tissues

To explore the clinical importance of miR‐375 in TSCC, we measured the miR‐375 expression in TSCC cell lines and samples using qRT‐PCR. We firstly revealed that the expression of miR‐375 was down‐regulated in the TSCC cell lines (SCC4, UM1, Cal27 and SCC1) compared with normal keratinocyte cell (NHOK) (Figure 4A). Next, we showed that the miR‐375 expression was lower in the TSCC tissues than in the adjacent normal tissues (Figure 4B). In addition, we found that the expression of miR‐375 was down‐regulated in 28 patients (28/40, 70%) compared with adjacent normal tissues (Figure 4C). Furthermore, we indicated that the lower miR‐375 expression was associated with the poor OS among the TSCC cohort by using Kaplan‐Meier survival analysis (Figure 4D). We also demonstrated that expression of miR‐375 was inversely correlated with LUCAT1 expression in the TSCC tissues (Figure 4E).

### 3.5 Knockdown expression of miR‐375 promoted TSCC cell proliferation, cycle and migration

**FIGURE 5:**
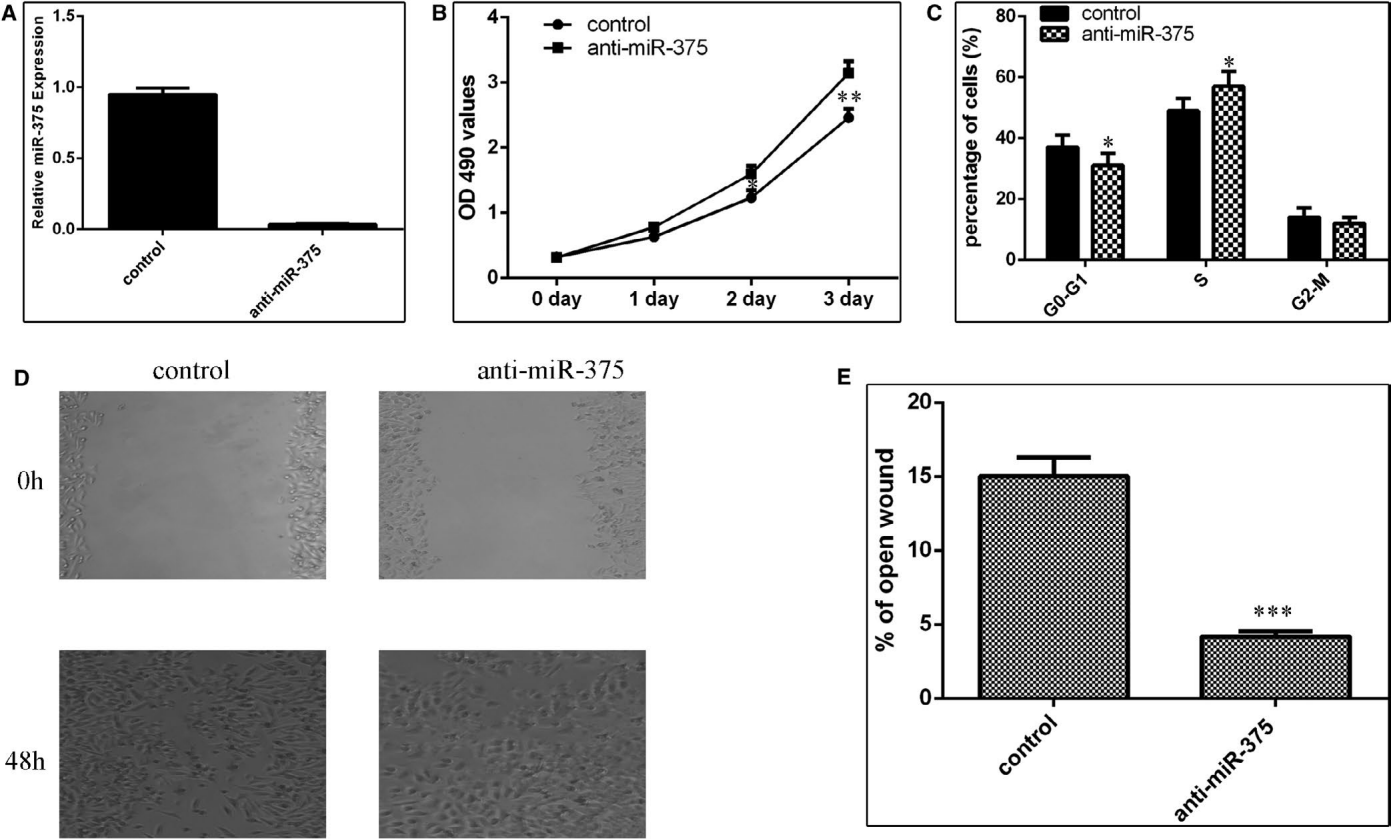
Knockdown expression of miR‐375 promoted TSCC cell proliferation, cell cycle and migration. (A) The expression of miR‐375 was measured by qRT‐PCR analysis. U6 was used as the internal control. (B) Knockdown expression of miR‐375 enhanced the SCC1 cell proliferation by using MTT analysis. (C) Inhibition expression of miR‐375 increased the S phases in the SCC1 cell. (D) Knockdown expression of miR‐375 enhanced the SCC1 cell migration. (E) The relative open wound was shown. *P < 0.05, **P < 0.01 and ***P < 0.001

TSCC cell line SCC1 cell was transfected with miR‐375 inhibitor (anti‐miR‐375) and negative control by using Lipofectamine 2000 (Figure 5A). Knockdown expression of miR‐375 enhanced the SCC1 cell proliferation by using MTT analysis (Figure 5B). Moreover, inhibition expression of miR‐375 increased the S phases in the SCC1 cell (Figure 5C). Wound scratch assay revealed that miR‐375 silencing promoted the SCC1 cell migration (Figure 5D and E).

### 3.6 LUCAT1 interacts with miR‐375 to regulate TSCC cell proliferation, cell cycle and migration

**FIGURE 6:**
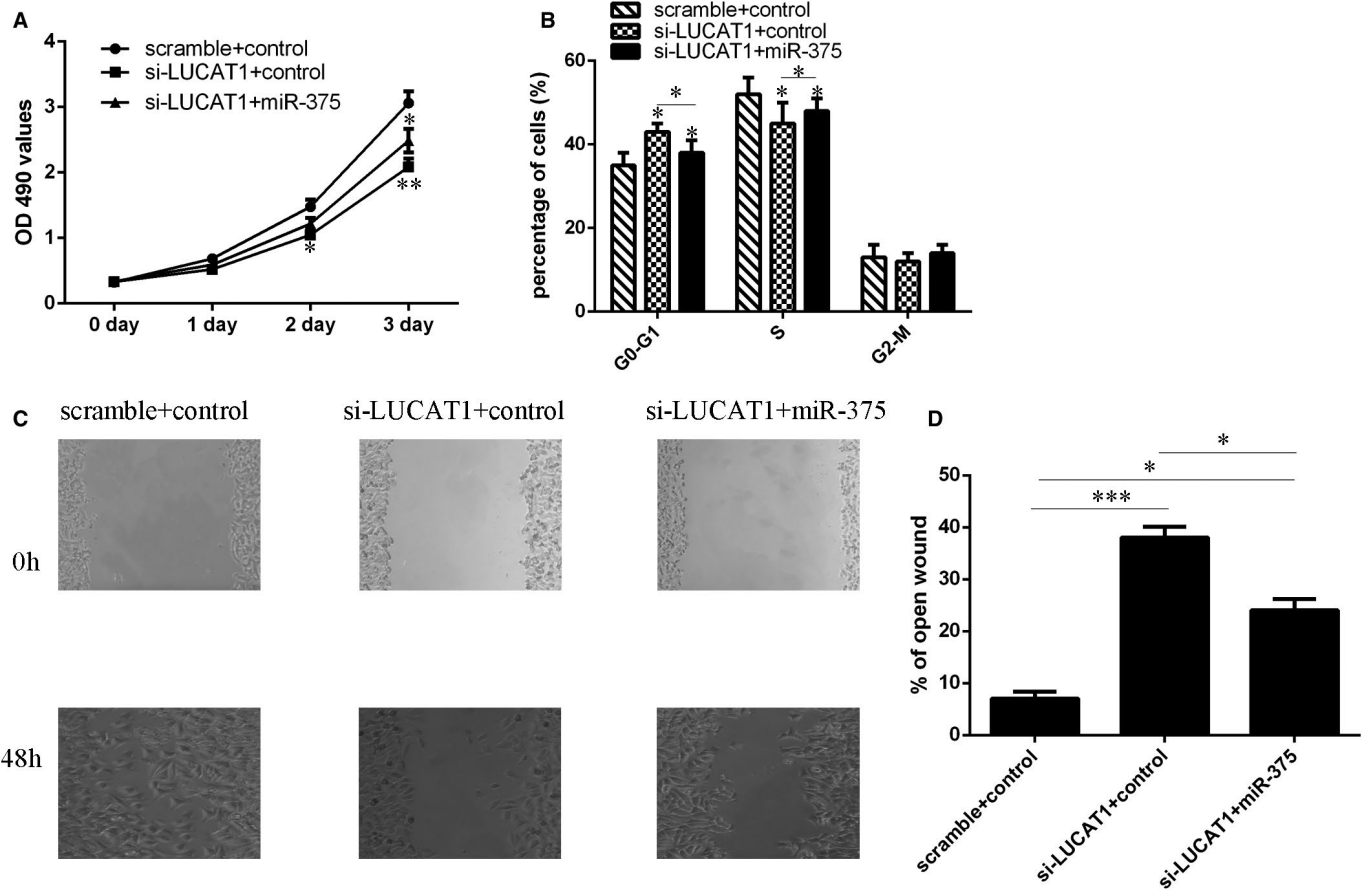
LUCAT1 interacts with miR‐375 to regulate TSCC cell proliferation, cell cycle and migration. (A) The cell proliferation in the different group was measured by MTT assay. (B) The cell cycle was determined by FACScan flow cytometer. (C) Wound scratch assay revealed that LUCAT1 silencing suppressed the SCC1 cell migration, whereas miR‐375 knockdown partially rescued the reduction in cell migration. (D) The relative open wound was shown. *P < 0.05, **P < 0.01 and ***P < 0.001

To further study the interaction between miR‐375 and LUCAT1, functional rescue experiments were done. As shown in Figure 6A, MTT assay indicated that LUCAT1 silencing suppressed SCC1 cells growth, whereas miR‐375 silencing partially rescued the reduction of growth. Moreover, knockdown expression of miR‐375 partially reversed the cell cycle suppression of LUCAT1 knockdown (Figure 6B). Furthermore, wound scratch assay revealed that LUCAT1 silencing suppressed SCC1 cell migration, whereas miR‐375 knockdown partially rescued the reduction of cell migration (Figure 6C and D).

## 4 DISCUSSION

TSCC is one of most common malignant cancers worldwide.^34^, ^35^ Cancer metastasis was the first cause of death in tumour patients, and searching novel biomarkers and elucidating the molecular mechanism of TSCC are the major topics of research on TSCC.^11^, ^36^, ^37^ Dysregulated expression of lncRNAs was found in a lot of human cancers using RNA sequencing and was correlated with tumour progression, survival and tumorigenesis in TSCC.^38^, ^39^, ^40^ We studied the role of a novel lncRNA LUCAT1 in the development of TSCC.

The lncRNA, LUCAT1, was reported to be up‐regulated in several tumours such as lung tumour, glioma, osteosarcoma, renal carcinoma and ESCC.^29^, ^30^, ^31^, ^32^, ^33^, ^41^ For example, Sun et al^33^ indicated that LUCAT1 expression was overexpressed in non–small cell lung cancer tissues and LUCAT1 knockdown suppressed the cell growth in vitro and in vivo. Gao et al^32^ found that the expression of LUCAT1 was up‐regulated in glioma cell lines and specimens. LUCAT1 knockdown decreased the glioma cell invasion and proliferation partly through regulating the miR‐375 expression. Han et al^31^ showed that LUCAT1 expression was overexpressed in the methotrexate (MTX)‐resistant cells and knockdown expression of LUCAT1 suppressed the osteosarcoma cell proliferation, tumour growth and invasion and some drug resistance‐correlated genes (MRP5, MDR1LRP1) partly through sponging miR‐200c expression. Yoon et al^30^ showed that the LUCAT1 expression was overexpressed in oesophageal squamous cell carcinoma (ESCC) tissues and cell lines. Knockdown expression of LUCAT1 inhibited the ESCC cell growth and promoted cell apoptosis through regulating the DNMT1 stability. Xiao et al^29^ indicated that LUCAT1 expression was up‐regulated in the renal carcinoma specimens and overexpression of LUCAT1 increased cell proliferation. However, role of LUCAT1 in the TSCC was still unknown. We firstly measured the expression of LUCAT1 in the TSCC cell lines and tissues. We demonstrated that the expression of LUCAT1 was up‐regulated in the TSCC cell lines and tissues and the higher LUCAT1 expression was associated with the poor overall survival. Knockdown expression of LUCAT1 suppressed TSCC cell proliferation, cycle and migration. These data suggested that lncRNA LUCAT1 may play as an oncogene role in the development of TSCC.

Growing evidence proved that a new regulatory mechanism exists between miRNAs and lncRNAs.^27^, ^42^, ^43^ LncRNA could play as molecular sponges to miRNA, thereby inhibiting miRNA expression.^22^, ^44^, ^45^ For examples, Chen et al^46^ demonstrated that lncRNA UICLM enhanced the TSCC liver metastasis through playing as a ceRNA for the miR‐215 expression to modulate ZEB2 expression. Xie et al^47^ showed that lncRNA ZFAS1 promoted the TSCC cell invasion and proliferation through acting as a ceRNA for miR‐484 expression. In addition, it has been shown that LUCAT1 increased the glioma cell invasion and proliferation through regulating miR‐375 expression. In this study, we showed that overexpression of miR‐375 decreased the luciferase activity of LUCAT1 wild‐type but not the LUCAT1 mutant type. Moreover, ectopic expression of miR‐375 suppressed the LUCAT1 expression and LUCAT1 silencing promoted the miR‐375 expression in the SCC1 cell. In addition, we demonstrated that miR‐375 expression was down‐regulated in the TSCC cell lines and tissues and the lower expression of miR‐375 was associated with poor OS. Furthermore, the expression of miR‐375 was inversely correlated with LUCAT1 expression in the TSCC tissues. Knockdown expression of miR‐375 promoted TSCC cell proliferation, cell cycle and migration. Knockdown LUCAT1 promoted the TSCC cell proliferation, cell cycle and migration partly through regulating miR‐375 expression.

In conclusion, our results reveal that LUCAT1 was up‐regulated in the TSCC cell lines and tissues and the higher LUCAT1 expression was associated with the poor overall survival. Knockdown expression of LUCAT1 suppressed TSCC cell proliferation, cell cycle and migration via sponging miR‐375 expression, providing a new insight into carcinogenesis of TSCC.

## CONFLICT OF INTEREST

The authors have no conflict of interest to declare.
